# Super-refractory status epilepticus in adults

**DOI:** 10.1186/s42466-022-00199-4

**Published:** 2022-08-22

**Authors:** Michael P Malter, Janina Neuneier

**Affiliations:** Department of Neurology, Faculty of Medicine, University Hospital Cologne, University of Cologne, Kerpener Straße 62, 50937 Cologne, Germany

**Keywords:** Status epilepticus, Super-refractory status epilepticus, Epilepsy, Standard operating procedure (SOP), Seizures, Standard operating procedure

## Abstract

**Introduction:**

Super-refractory status epilepticus (SRSE) represents the culmination of refractory status epilepticus (RSE) and carries a significant risk of poor neurological outcome and high mortality. RSE is not defined primarily by seizure duration, but by failure to respond to appropriate antiseizure treatment. SRSE is present when a RSE persists or recurs after more than 24 h of treatment with anesthetics. No evidence-based treatment algorithms can be provided for SRSE. Therefore, we propose a pragmatic standard operating procedure (SOP) for the management of SRSE that addresses the existing uncertainties in the treatment of SRSE and provides options for resolution and decision-making.

**Comments:**

First, we recommend the assessment of persistent seizure activity and the evaluation of differential diagnoses to confirm correct diagnosis. Relevant differential diagnoses include psychogenic non-epileptic seizures, hypoxic, metabolic, or toxic encephalopathies, and tetanus. During SE or in severe encephalopathies, a so-called electroclinical ictal-interictal continuum may occur, which denotes an intermediate stage that cannot be defined with certainty as ictal or interictal by EEG and should not lead to harmful overtreatment. Because both prognosis and specific treatment options depend crucially on the etiology of SRSE, the etiological evaluation should be performed rapidly. When SRSE is confirmed, various pharmacological and non-pharmacological treatment options are available.

**Conclusion:**

We provide a pragmatical SOP for adult people with SRSE.

## Introduction

Status epilepticus (SE) signifies an abnormally prolonged seizure activity due to either a failure of seizure-limiting mechanisms or an exaggeration of seizure-aggravating mechanisms with low chance of self-limitation, and thus requiring prompt recognition and management [1]. Super-refractory status epilepticus (SRSE) represents the climax of therapy refractory status epilepticus (RSE).

Relevant semiological criteria in SE are the presence of motor signs or an impaired consciousness. Motor signs can be focal or generalized convulsions. SE without prominent motor signs is commonly referred to as non-convulsive SE (NCSE). NCSE can be further categorized as focal or generalized. Within the latter category, typical absence SE represents the most benign SE form with good therapy response and prognosis, as it can be easily interrupted in most cases with moderate doses of benzodiazepines and non-sedating ASM that are effective against generalized seizures, especially valproate and levetiracetam. To identify typical absence SE, the typical EEG pattern with generalized spike-wave paroxysms at a frequency of 3/s or the knowledge of an existing absence epilepsy is required. It should be noted that in SE there is often no uniform but evolutionary semiology over the entire period. Pragmatically, the worst seizure manifestation should be used for categorization and management in decision making, i.e., if generalized convulsions occur at any time during SE, a convulsive SE should be assumed.

Drug treatment algorithms in SE include a three-step approach [2]. In the first step, benzodiazepines are drugs of choice, followed in the second step by intravenous antiseizure medications (ASM), such as phenytoin/fosphenytoin, valproate, or levetiracetam, and to a lesser extent but with growing evidence, lacosamide and brivaracetam. In the third step, anesthetics are used, which includes intubation, ventilation, and ICU admission. Both the appropriate dosage of each drug and its rapid application are crucial for termination of SE.

The incidence of SE in Europe is 10-30/100.000 [2]. Higher age and drug-resistance are risk factors for poor outcome and death, whereas ASM-withdrawal or alcohol-related SE are etiologies with presumed favorable outcome [3]. Up to 48% of patients with SE progress to RSE, and 22% of patients with RSE progress into SRSE [4]. SRSE in particular has a substantial risk for poor neurological outcome with mortality of 35% [4]. Most SRSE occur de novo, i.e. without pre-existing epilepsy. In addition to the direct impact of SE on neuronal structures leading to neuronal damage, the complication rate increases with the duration of intensive care unit (ICU) -treatment with mechanical ventilation.

Here, we attempt to establish a pragmatical standard operating procedure (SOP) for the management of adult people with SRSE.

### Definition

SRSE is not primarily defined by seizure duration, but by failure to respond to appropriate antiseizure treatment. A refractory SE (RSE) is present when first- and second-line treatments have failed, whereas a SRSE is presumed when a SE persists or recurs after treatment with anesthetics for more than 24 h [2, 4].

## First steps

SE and especially SRSE are critical neurological emergencies. Therefore, treatment should be provided in specialized units with high expertise in neuro-intensive care. In addition to established general ICU standards of care, we would like to highlight here only special procedures for people with SE:

- Organization of sufficient intensive care capacity with the possibility of ventilation even before the patient is admitted.
- No holding, no biting wedge during convulsive seizures.
- If chronic alcohol abuse is present: thiamine 100 mg i.v.
- If serum glucose < 60 mg/dl: glucose 40% 60 ml (*after* thiamine administration).

### Note

In the absence of thiamine, glucose is increasingly degraded to lactate; addition of glucose alone increases lactic acidosis with high risk of Wernicke's encephalopathy. Therefore, thiamine should be administered first, followed by glucose.

## EEG

EEG is one of the most important investigations in the management of SE, both for diagnosis and treatment evaluation. Up to 20% of SE with clinically evident seizures progress to a non-convulsive form, which cannot be adequately assessed without EEG. EEG interpretation in SE is not trivial. In addition to the difficulty in clearly distinguishing ictal and interictal patterns, sedating ASM, especially anesthetics, can produce changes in EEG patterns that can be confusing. Therefore, EEG interpretation should be performed only by experienced and certified interpreters to avoid misdiagnosis and overtherapy.

In recent years, continuous EEG monitoring (CEEG) has become more widely used in ICUs, but comprehensive standards for its application and documentation are still lacking. Although CEEG improves detection of subclinical and non-convulsive seizures and SE in critical ill patients, it has not yet shown improvement in clinical outcome. Therefore, in absence of CEEG capacity, repetitive EEG can be used further as an alternative.

## Comments/explanations/additions (see footnotes in the flowchart, Fig. 1)

We propose an algorithm that both addresses existing uncertainties in the management of SRSE for treating physicians and highlights established resolution and decision options.

**Fig. 1:**
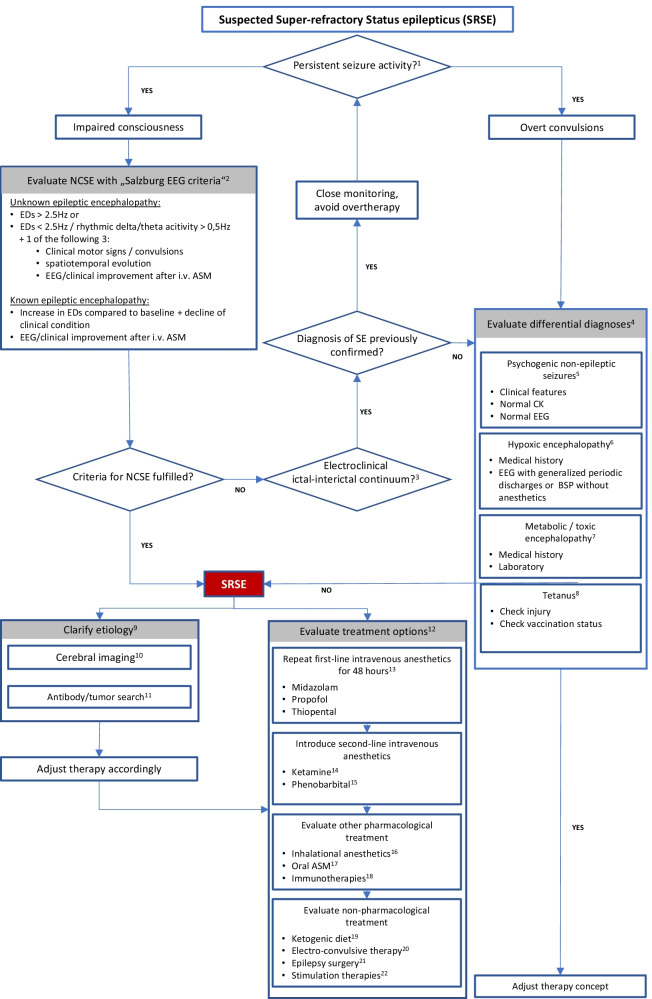
Flowchart of the  proposed algorithm for the management of SRSE. Abbreviations: EEG: electroencephalography; NCSE: non-convulsive status epilepticus; ED: epileptiform discharges on EEG; i.v.: intravenous; ASM: antiseizure medication; SRSE: super-refractory status epilepticus; CK: creatine kinase; BSP: burst-suppression pattern on EEG

1. Clinical seizure semiology is crucial for the assessment of persistent seizure activity. While overt convulsions can more easily lead to a correct diagnosis, in NCSE there are neither specific clinical seizure signs or symptoms for diagnosis nor definite EEG consensus criteria. In particular, the distinction between generalized periodic EEG discharges in hypoxic, metabolic or toxic encephalopathies and epileptiform patterns can lead to diagnostic uncertainty and often requires the inclusion of additional clinical parameters in the assessment.
2. To overcome the existing diagnostic deficiencies for NCSE, the "Salzburg EEG criteria" were developed as a combination of EEG and clinical parameters to facilitate the diagnosis of NCSE [6].
3. In quite a few cases, a so-called electroclinical ictal-interictal continuum can be found in EEG [5]. This is an intermediate state that cannot be defined with certainty as ictal or interictal and causes corresponding uncertainty as to whether therapy must be intensified or a wait-and-see approach is advisable in order to avoid harmful overtreatment. If a previously confirmed SE has an electroclinical ictal-interictal continuum without clear clinical signs of persistent seizures, close wait-and-see monitoring is more advisable than escalation of therapy. In case of uncertain diagnosis of SE differential diagnoses should be evaluated.
4. Differential diagnoses of SRSE should always be carefully considered to avoid misdiagnosis and overtreatment.
5. In particular, the longer duration of psychogenic non-epileptic seizures (PNES) compared to epileptic seizures and the unresponsiveness to antiseizure treatment carry a high risk of being misinterpreted as SE. Clinical diagnosis of PNES is challenging because there is no single seizure sign or symptom that reliably and fundamentally distinguishes a PNES from an epileptic seizure. Clinical features suggestive of PNES include:
  Closed eyes and especially active blinking when eyes are passively opened
  Lateral head bobbing
  Asynchronous convulsive movements of the extremities with myoclonus of the arms and legs at different frequencies
  Opisthotonus with pelvic thrusting
  Rotations around the longitudinal axis of the body
  Fluctuating course with movement modulation altered by distraction or pain stimuli
  Undulated impairment of consciousness and responsiveness.
  Injury, tongue biting, enuresis, and cyanosis may also occur in PNES [7]. Unlike SE, no epileptic EEG seizure patterns are found in PNES. Creatine kinase (CK) is not elevated in PNES except after injury. To date, there are no cut-off values that clearly distinguish CK elevation due to seizure-related muscle activity from injury-related changes. As a rule of thumb, a CK elevation greater than 1000 U/l is highly suspicious for a seizure-related cause. It should be noted that CK elevation may occur with a delay of 12 h, with peak values reached after 24–72 h [8]. The determination of further laboratory parameter to confirm or exclude epileptic seizures should be reserved for specialized institutions because of the high risk of false positive and false negative findings and thus erroneous interpretations.
6. Hypoxic encephalopathy (HE) is typically characterized by an initial coma with development of stimulus-sensitive myoclonus during its course; indicative factors include medical history (e.g., initial coma, asystole, cardiopulmonary resuscitation), concomitant circumstances (e.g., acute coronary syndrome, pulmonary embolism), and an EEG with evidence of typical generalized periodic discharges or a burst suppression pattern in the absence of administered sedatives or anesthetics. Although HE is widely believed to be pathophysiologically distinct from SE, irritatingly, patients with HE are regularly included in reports of SE treatment, complicating outcome assessment of SE.
7. In metabolic or toxic encephalopathies, the main symptoms here are decreased vigilance. Myoclonus and tremor and asterixis may occur but are not prominent. Metabolic disorders and laboratory findings suggest hepatic, uremic or septic constellations.
8. Tetanus occurs only very sporadically in industrialized countries today due to vaccination campaigns. The clinical picture is characterized by toxin-related neurological signs with increased muscle tone (incl. trismus and ophisthotonus) and convulsions that can be confused with a convulsive SE. Differentiation is possible by EEG because there are no typical epileptic patterns in tetanus.
9. Since prognosis and specific treatment options depend critically on the etiology of SE, etiologic clarification should be rapid when SRSE is confirmed. Because of time-dependent changes in neurotransmission (see Comment 12), in which responsiveness to treatment changes rapidly over time, diagnostic efforts must be conducted without loss of time to treatment.
10. While in the acute situation computed tomography (CT) may be sufficient or only possible to identify acutely treatable causes, magnetic resonance imaging (MRI) should be performed in the later course of SE due to its higher sensitivity. Relevant pathologies for treatment decisions are evidence of a focal epileptogenic lesion or encephalitic abnormalities. It should be noted that imaging pathologies may also be caused by sustained seizure activity itself. SE-associated MRI changes are often focal signal enhancements in FLAIR-, T2- and diffusion-weighted sequences, not necessarily in the seizure onset zone, particularly in the cortex, which usually regress over weeks after SE. In addition, these changes are also found in subcortical regions such as the basal ganglia and pulvinar thalami, medial temporal structures, and cerebellum [9]. It can be difficult to distinguish these SE-related changes from acute stroke signs. When in doubt, performing perfusion CT may help in the differential diagnostic evaluation: Ictal changes may result in regional hyperperfusion that often extends beyond the corresponding vascular territory, whereas in ischemic stroke, there is regional hypoperfusion restricted to vascular territories.
11. Autoimmune-mediated encephalitis is a rapidly growing topic with a heterogeneous clinical picture. Currently, it is unknown how many patients with SRSE have an underlying autoimmune cause, but also conversely, how many patients with autoimmune encephalitis are misdiagnosed as having SRSE. The term New Onset Refractory SE (NORSE) was introduced for patients with RSE without a clear acute structural, toxic, or metabolic cause and without known history of seizures or known neurological disease. The most common etiologies identified in NORSE were autoimmune (19%) and paraneoplastic (18%) encephalitis. The most prevalent antibodies found in NORSE were directed against surface antigens such as the N-methyl-D-aspartate (NMDA) receptor, leucine-rich glioma inactivated protein 1 (LGI1) and γ-aminobutyric-acid-B receptor (GABABR), other antibodies were found in varying frequencies [10]. Nevertheless, we recommend a complete antibody panel search in CSF and serum and tumor search to avoid overlooking rare causes. Conversely, each finding should be checked for plausibility.
12. As outlined above, there are no evidence-based treatment algorithms for SRSE, but several pharmacological and non-pharmacological treatment options have become established in clinical management. The rationale behind aggressive treatment of SRSE is to prevent irreversible neuronal damages with relevant long-term sequelae. Such consequences are most obvious in SE with generalized convulsions and unknown in other SE types, Therefore, NCSE should be treated predominantly with non-sedating ASM. Understanding the time-dependent changes in neurotransmission during sustained seizure activity may help to understand the rationale for treatment decisions. After SE onset, there is a progressive internalization of inhibitory GABA-receptors with a concomitant increase in excitatory NMDA-receptors in the postsynaptic membrane. Therefore, GABA-ergic substances should be used predominantly in early SE, whereas substances acting against NMDAR-receptors may be more useful in later SE. In clinical management, RSE/SRSE rapidly results in gradual accumulation of agents without concomitant reduction of ineffective medications. Therefore, it should always be evaluated which medications have not provided benefit and should therefore be discontinued quickly to avoid drug side effects causing further complications and confusion.
13. Anesthetics are agents of choice for RSE, and recommended agents and dosages [2] are listed in Table 1. The treatment goal under anesthetics is absence of clinical or electroencephalographic seizure equivalents or an EEG burst suppression pattern for at least 24 h, requiring ICU admission with intubation and mechanical ventilation. Recommended first-line agents are midazolam, propofol and thiopental, second-line anesthetics are ketamine and phenobarbital. There is no comparative evidence for the superiority of one particular anesthetic over another, nor for combination strategies, nor for the duration of application and reduction procedures. If the first chosen anesthetic fails to permanently suppress seizure activity in RSE results in SRSE.

**Table 1:** Dosages of intravenous anesthetics, adopted and modified from [2]

1. Midazolam: 0.2 mg/kg BW i.v. as bolus, *pragmatically*: ≥ 50 kg BW: 10 mg, ≥ 70 kg BW 14 mg, ≥ 100 kg BW 20 mg, maintenance dose ca. 0.1–0.5 mg/kg/h for 24 h
2. Propofol: 2 mg/kg BW i.v. as bolus, *pragmatically:* ≥ 50 kg BW: 100 mg, ≥ 70 kg BW 140 mg, ≥ 100 kg BW200 mg, maintenance dose 4–10 mg/kg/h for 24 h
3. Thiopental: 5 mg/kg BW i.v. as bolus, *pragmatically:* ≥ 50 kg BW: 250 mg, ≥ 70 kg BW 350 mg, ≥ 100 kg BW 500 mg, maintenance dose ca. 3–7 mg/kg/h for 24 h
4. Ketamine: 2 mg/kg i.v. as bolus, *pragmatically*: ≥ 50 kg BW: 100 mg, ≥ 70 kg BW 140 mg, ≥ 100 kg BW 200 mg, maintenance dose 1–7 mg/kg/h for 24 h, to prevent nightmares as side effect always in combination with midazolam according to 1
5. Phenobarbital: 15–20 mg/kg BW i.v., max. 100 mg/min, target serum levels: 30–50 µg/ml

*mg* milligram; *kg* kilogram; *BW* body weight; *h* hour; *µg* mikrogram; *ml* milliliter

If the treatment goal cannot be achieved with the first chosen anesthetic, you may change the agent or introduce combination therapy. If SE recurs after discontinuation of the anesthetic, one treatment option is to reintroduce of selected anesthetic for a second longer cycle of 48 hours or switch to a different anesthetic.
14. Ketamine acts as a non-competitive NMDA-receptor antagonist. Since excitatory NMDA-receptors are increasingly upregulated with sustained seizure activity, the mechanism of action may be particularly effective in the later course of SE. Another relevant advantage of ketamine is the absence of cardiopulmonary depression, so that the use of catecholamines may be not necessary. To avoid nightmares as a relevant side effect of ketamine, the additional administration of midazolam is strongly recommended, which has synergistic effects on the duration of action of ketamine.
15. Phenobarbital, which is here classified in the group of anesthetics but can also be counted among the ASM, has somewhat fallen out of focus in clinical practice due to its side effect profile and limited tolerability for the treatment of SE. Nevertheless, its good antiseizure effect is undisputed and a recent review comparing the efficacy of different ASM found phenobarbital to be the most effective i.v. ASM [11]. Relevant interactions with valproate, which acts as an inhibitor of liver enzymes and therefore slows the metabolization of phenobarbital, must be considered.
16. Inhalational anesthetics have shown good efficacy in immediate seizure control, but high relapse rates after discontinuation. In particular, isoflurane has emerged as a potential new rescue therapy option for RSE/SRSE. With the AnaConDa^®^-system, long-term use outside the operating room became feasible on ICU. In a recent retrospective multicenter study with 45 patients who received isoflurane for treatment of RSE/SRSE, RSE/SRSE was terminated in 23/45 patients (51%) of the total group and in 13/45 patients (29%) without additional therapy [12].
17. Despite a number of reports on the use of oral ASM in RSE and SRSE, the available data do not allow a clear conclusion on the extent to which they are effective in SRSE. Maybe, the additional administration of an oral ASM in SRSE may serve to prevent seizure recurrence after discontinuation of anesthetics.
18. Immunotherapy represents the mainstay of treatment for autoimmune encephalitis. First-line regimens consist of high-dose cortisone pulses (1000 mg methylprednisolone per day for 3 to 5 consecutive days) and intravenous immunoglobulins (0.4 kg/kg for 5 consecutive days) or, if direct pathogenic antibodies to surface antigens are detected, apheresis therapies. Second-line regimens consist of rituximab and cyclophosphamide [13].
19. The ketogenic diet (KD) represents a high-fat, low-carbohydrate diet with good antiseizure effects in children and adults [14]. KD can be applied via enteral feeding tube or intravenously. Therapeutic goal is to achieve a ketotic metabolism. Concurrent administration of propofol is considered a relative contraindication because of the increased risk of propofol infusion syndrome. Therefore, propofol must be discontinued 24 h prior to initiation of KD.
20. In a systematic review, 19 patients were identified treated with electroconvulsive therapy in SRSE, a therapeutic response was seen in 58% of cases. It can therefore be evaluated as rescue therapy in SRSE [15].
21. Epilepsy surgery may be considered in focal SRSE. Resective and disconnective surgical procedures have been reported in SRSE with varying degrees of success. Eligible candidates for resective surgery are those in whom a definite epileptogenic lesion is detected. It should be kept in mind that resective surgery is irreversible and may damage eloquent brain areas. Therefore, we recommend these treatment options only be performed in a specialized epilepsy surgery center. We do not recommend disconnective procedures such as multiple subpial transections and callosotomy due to uncertain efficacy.
22. Regarding stimulation therapies for SE, there are very preliminary data for deep brain stimulation (DBS) and vagus nerve stimulation (VNS). Because these treatments are invasive long-term procedures with associated risks, we recommend that they be applied only applied in the context of studies for SRSE.

## Approaches with negative prospective study data

Data from a randomized, multicenter, controlled trial of 268 SE patients on mild hypothermia, aiming for a target body temperature of 33 °C, showed no effect in SRSE [16].

Brexanolone (Allopregnanolone) was equally effective to terminate SRSE than placebo in a randomized double-blinded, placebo-controlled, phase-3 study trial, so that it cannot be recommended for the treatment of SRSE (ClinicalTrials.gov:NCT02477618).

## Conclusions

SRSE represents the most severe manifestation of SE and carries a significant risk of poor neurologic outcome and mortality. We propose a pragmatic SOP addressing options for the treatment of SRSE. Randomized controlled trials are needed to improve evidence-based diagnostic and therapeutic decision making.

## Data Availability

Not applicable.

## Abbreviations

ASM: Antiseizure medication
BW: Body weight
CK: Creatine kinase
CT: Computed tomography
DBS: Deep brain stimulation
EEG: Electroencephalography
GABABR: Gamma-amino-butyric acid type B receptor
HE: Hypoxic encephalopathy
ICU: Intensive care unit
Kg: Kilogram
KD: Ketogenic diet
LGI1: Leucine-rich-glioma-1 protein
l: Liter
ml: Milliliter
MRI: Magnet resonance imaging
NCSE: Non-convulsive status epilepticus
NMDA: N-methyl-D-aspartate
NORSE: New onset refractory status epilepticus
PNES: Psychogenic non-epileptic seizure
SE: Status epilepticus
SOP: Standard operating procedure
SRSE: Super-refractory status epilepticus
RSE: Refractory status epilepticus
U: Units
VNS: Vagus nerve stimulation
